# Supplementary material for: Physical Activity and Frailty Are Impaired in Older Adults with Benign Paroxysmal Positional Vertigo
Source: J Clin Med. 2024 Dec 11;13(24):7542. doi: 10.3390/jcm13247542 (PMC11728343; doi:10.3390/jcm13247542)
Supplement: Supplementary file 1 [file jcm-13-07542-s001.zip › jcm-3326900-supplementary.pdf]

Supplementary Materials

Supplementary Table S1. Required Cohen's D according to sample size and statistics.

Outcome	N		Cohen's d at 80% power α= 0.05		
	oaBPPV	CON	T-test	Mann-Whitney U	Chi-square
MOX	32	22	0.7	0.71	/
IPAQ & subjective well-being	37	22	0.67	0.69	0.51
Frailty	36	21	/	/	0.51

Supplementary Table S2: Results of sub-analyses on subject characteristics.

Subject characteristics	$oaBPPV_{frail}$	$oaBPPV_{robust}$	$control_{robust}$	P-value	Post-hoc comparison *		
					$oaBPPV_{frail}$ vs. $oaBPPV_{robust}$	$oaBPPV_{frail}$ vs. $control_{robust}$	$oaBPPV_{robust}$ vs. $control_{robust}$
N (F/M)	11 (8/3)	10 (4/6)	17 (9/8)	0.31			
Age	72.5(4.7)	71.8(4)	74.2(4.5)	0.37			
Weight (kg)	75.9(13.6)	76.8(11.5)	75.1(8.7)	0.93			
Height (m)	1.7(0.1)	1.7(0.7)	1.7(0.1)	0.07			
BPPV							
RPSCC (n)	5	7	0				
LPSCC (n)	6	2	0				
Bilateral PSCC (n)	0	0	0	**<.001**			
RLSCC geotropic(n)/apogeotropic (n)	0	0	0				
LLSCC geotropic (n)/ apogeotropic (n)	0	1/0	0				
No BPPV (n)	0	0	17				
Duration of complaints							
Some days (n)	1	1	0				
Several weeks (n)	0	2	0	**<.001**			
Several months (n)	10	7	0				
No complaints (n)	0	0	17				
Walking aid				0.27			
None (n)	9	10	17				

Crutch (n)<br>Walker (n)	1<br>1	0<br>0	0<br>0				
Sleeping pattern<br>Good (n)<br>Restless (n)<br>Long time needed to fall asleep (n)<br>Restless+ long time needed (n)	 6<br>2<br>0<br>3	 4<br>4<br>2<br>0	 12<br>2<br>3<br>0	**0.04**			
Number of comorbidities	2(1-5)	2.5(1-4)	2(0-5)	0.5			
Number of medications	7(0-10)	3(1-6)	2(0-8)	**0.01**	**0.04**	**0.01**	0.1
MOCA total score	22(16-29)	23(18-29)	28(23-30)	**0.008**	0.18	**0.01**	**0.002**

*Post-hoc comparison adjusted for multiple testing with Tukey after one-way ANOVA and Bonferroni after Kruskall Wallis. Significant differences are indicated in bold. Normally distributed data are expressed as mean (SD), non-normally distributed data as median (minimum-maximum).

Abbreviations: oaBPPV$_{frail}$*, frail older adults with BPPV; oaBPPV*$_{robust}$*, robust older adults with BPPV; control*$_{robust}$*, robust older adults in control group; F, female; M, male; BPPV, benign paroxysmal positioning vertigo; RPSCC, right posterior semicircular canal BPPV; LPSCC, left posterior semicircular canal BPPV, RLSCC, right lateral semicircular canal BPPV; LLSCC, left lateral semicircular canal BPPV; MOCA, Montreal Cognitive Assessment scale.*

Supplementary Table S3: Results of sub-analyses on subjective well-being.

Subjective well-being	$oaBPPV_{frail}$	$oaBPPV_{robust}$	$control_{robust}$	P-value	Post-hoc comparison *		
					$oaBPPV_{frail}$ vs. $oaBPPV_{robust}$	$oaBPPV_{frail}$ vs. $control_{robust}$	$oaBPPV_{robust}$ vs. $control_{robust}$
DHI total score	38(20-74)	22(10-62)	0(0-10)	**<.001**	**0.01**	**<.001**	**<.001**
DHI physical subscale	16(8-24)	13(6-22)	2(0-5)	**<.001**	0.08	**<.001**	**<.001**
DHI emotional subscale	10(2-24)	4(2-16)	0(0-4)	**<.001**	0.03	**<.001**	**<.001**
DHI functional subscale	16(8-30)	8(0-24)	0(0-6)	**<.001**	**0.01**	**<.001**	**0.001**
FES-I	34(19-53)	21(17-38)	18(8-31)	**<.001**	**0.01**	**<.001**	**0.01**
GDS-15	4(0-13)	1(0-5)	1(0-4)	**<.001**	**0.01**	**<.001**	0.39

*Post-hoc comparison adjusted for multiple testing with Tukey after one-way ANOVA and Bonferroni after Kruskall Wallis. Significant differences are indicated in bold. Normally distributed data are expressed as mean (SD), non-normally distributed data as median (minimum-maximum).

Abbreviations: $oaBPPV_{frail}$, frail older adults with BPPV; $oaBPPV_{robust}$, robust older adults with BPPV; $control_{robust}$, robust older adults in control group; DHI, dizziness handicap inventory; FES-I, international falls efficacy scale; GDS-15, 15-item geriatric depression scale.

Supplementary Table S4: Results of sub-analyses on objective physical activity.

	$oaBPPV_{frail}$	$oaBPPV_{robust}$	$control_{robust}$	P-value*	Post-hoc comparison*		
					$oaBPPV_{frail}$ vs. $oaBPPV_{robust}$	$oaBPPV_{frail}$ vs. $control_{robust}$	$oaBPPV_{robust}$ vs. $control_{robust}$
Sedentary min/day	1182.5(88.2)	1083.7(130.4)	1069(112.2)	0.02			
Standing min/day	132.9(101.3-211.9)	184.93(90.3-480.7)	212.4(92.5-335.6)	0.08			
Dynamic min/day	105.8(52.6)	138(37.1)	160(52)	**0.01**	**0.01**	**0.01**	0.21
LPA min/day	83.8(51.8-189.3)	86.6(54.6-139.7)	96.7(53.5-165.1)	0.03			
MPA min/day	18.6(0.2-49.4)	39.5(21.6-136.8)	49.3(5.7-119.3)	**0.003**	**0.002**	**0.003**	0.27
VPA min/day	0(0-0)	0.3(0-8.7)	0.05(0-41.8)	**0.007**	**0.002**	**0.01**	0.25
Number of postural transitions	224.7(110.98)	204.80(77.387)	232(79.9)	0.34			
Number of dynamic bouts 5-10 min	0(0-1.5)	0.75(0-4.5)	0.75(0-3.5)	0.04			
Number of dynamic bouts ≥10 min	0(0-0.3)	0(0-1.75)	0.5(0-3)	0.08			
Number of sedentary bout ≥30 min	10.3(7.3-14.3)	11(5-13.3)	6.8(4.8-16)	**0.01**	0.32	**0.003**	**0.04**
Bout duration (min) of dynamic bouts ≥10 min	12(12-17.21)	19.9(10.6-23.9)	15.2(10.3-34.1)	0.16			

Bout duration (min) of sedentary bouts ≥30 min	60.5(49.9-77.7)	56.5(46-95.27)	55.9(44.3-105.1)	0.4			

*Post-hoc comparison adjusted for multiple testing with Tukey after one-way ANOVA and Bonferroni after Kruskall Wallis. Significant differences are indicated in bold. Significant differences are indicated in bold. Normally distributed data are expressed as mean (SD), non-normally distributed data as median (minimum-maximum).
Abbreviations: oaBPPV$_{frail}$, frail older adults with BPPV; oaBPPV$_{robust}$, robust older adults with BPPV; control$_{robust}$, robust older adults in control group; LPA, low physical activity; MPA, moderate physical activity; VPA, vigorous physical activity.

Supplementary Figure S1: Results of sub-analyses on physical activity pattern during day.

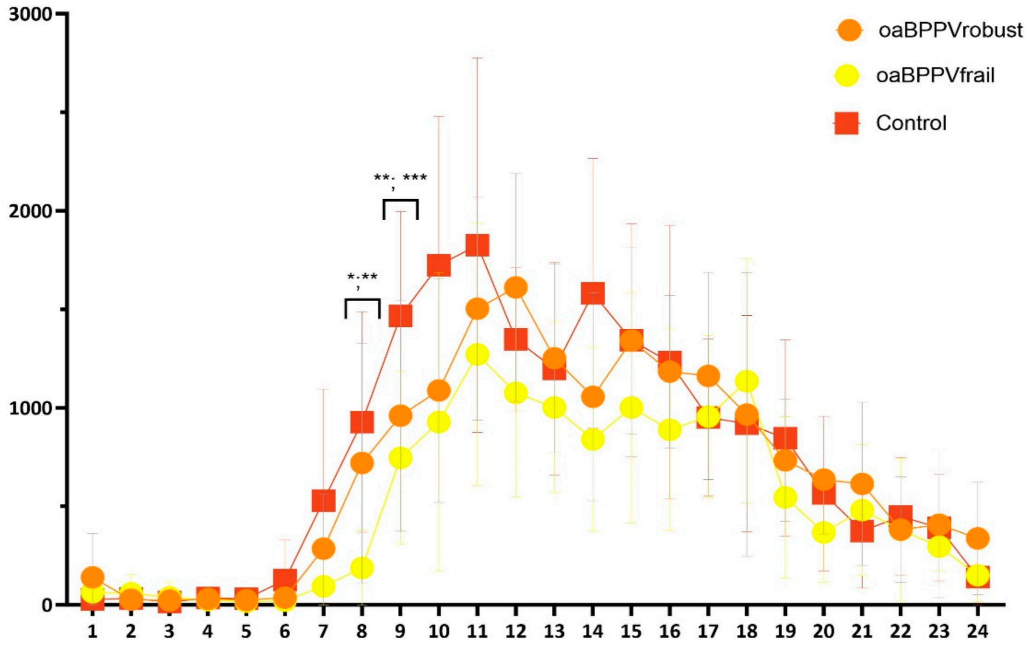

Supplementary Figure S1. Distribution of the mean physical activity intensities per 24 h without (A) and with (B) 1-hour time-shift in frail older adults with BPPV (n=) , and robust (n=) older adults witt BPPV and controls (n=). '*' indicates a significant difference between $oaBPPV_{frail}$ and $oaBPPV_{robust}$, '**' indicates a significant difference between $oaBPPV_{frail}$ and $control_{robust}$ and '***' indicates a significant difference between $oaBPPV_{robust}$ and $control_{robust}$. Normally distributed data are expressed as mean (SD), non-normally distributed data as median (minimum-maximum).

Abbreviations: $oaBPPV_{frail}$, frail older adults with BPPV; $oaBPPV_{robust}$, robust older adults with BPPV; $control_{robust}$, robust older adults in control group

Supplementary Table S5: results of sub-analyses on subjective physical activity.

	oaBPPV$_{frail}$	oaBPPV$_{robust}$	control$_{robust}$	P-value	Post-hoc comparison *		
					oaBPPV$_{frail}$ vs. oaBPPV$_{robust}$	oaBPPV$_{frail}$ vs. control$_{robust}$	oaBPPV$_{robust}$ vs. control$_{robust}$
**Categorical**							
Low (N)<br>Moderate (N)<br>Vigorous (N)	8<br>0<br>3	3<br>0<br>7	1<br>0<br>16	**<.001**			
**MET-minutes/ day**							
Work	0(0-0)	0(0-3360)	0(0-10350)	0.05			
Transport	0(0-396)	0(0-1965)	252(0-1260)	0.02			
Household	90(0-4020)	450(0-2280)	540(0-63420)	0.12			
Leisure	0(0-480)	601.6(0-1548)	1512(240-14697)	**<.001**	**0.002**	**<.001**	**0.001**
Walking	0(0-396)	330(0-1221)	792(0-6336)	**<.001**	**0.004**	**<.001**	**0.03**
Sitting	388.8(153.6)	393.3(95.9)	335(128.5)	0.24			
MPA	330(0-4020)	1155(0-4080)	3600(180-63720)	**0.005**	0.11	**0.002**	**0.03**
VPA	0 (0-0)	0(0-960)	0(0-9600)	0.11			

*Post-hoc comparison adjusted for multiple testing with Tukey after one-way ANOVA and Bonferroni after Kruskall Wallis. Significant differences are indicated in bold. Normally distributed data are expressed as mean (SD), non-normally distributed data as median (minimum-maximum).
Abbreviations: oaBPPV$_{frail}$, frail older adults with BPPV; oaBPPV$_{robust}$, robust older adults with BPPV; control$_{robust}$, robust older adults in control group; MET, metabovlic resting rate; MPA, moderate physical activity; VPA, vigorous physical activity.
